# Prefecture‐Level Prehospital Glucose Administration for Hypoglycemia and Diabetologist Density Among Older Adults in Japan: A Nationwide Ecological Study

**DOI:** 10.1111/ggi.70639

**Published:** 2026-07-09

**Authors:** Tomoya Saika, Yuki Kado, Michiko Yamazaki, Tohru Takebe, Masaya Sakamoto, Atsushi Nakagomi

**Affiliations:** ^1^ Patient Engagement Frontier Medical Science KAKEHASHI Inc. Tokyo Japan; ^2^ Graduate School of Medical and Pharmaceutical Sciences Chiba University Chiba Japan; ^3^ Department of Hematology Nippon Medical School Tokyo Japan; ^4^ Department of Diabetes, Metabolism & Endocrinology International University of Health and Welfare Mita Hospital Tokyo Japan; ^5^ Department of Diabetes, Metabolism & Endocrinology International University of Health and Welfare Narita Hospital Chiba Japan; ^6^ Department of Social Preventive Medical Sciences, Center for Preventive Medical Sciences Chiba University Chiba Japan

**Keywords:** diabetologist density, ecological study, Emergency Medical Services, geographic disparities, hypoglycemia

## Abstract

**Aims:**

To assess prefecture‐level disparities in prehospital glucose administration for hypoglycemia among older adults, diabetologist density, and insulin prescribing, and to examine the association between diabetologist density and prehospital glucose administration in Japan.

**Methods:**

We conducted a nationwide ecological cross‐sectional study across all 47 prefectures. Prehospital glucose administration for hypoglycemia was identified from national Emergency Medical Service statistics (2022). Diabetologist density (certified diabetologists per 100 000 population) was obtained from the Japan Diabetes Society specialist list. Prescribing patterns were derived from the Musubi electronic medication history database (January 2019 to June 2025) and summarized as the proportion of insulin prescriptions among glucose‐lowering drugs. Pearson correlation coefficients were calculated.

**Results:**

Rates of prehospital glucose administration for hypoglycemia varied substantially across prefectures (approximately 10‐fold between the highest and lowest). Diabetologist density was inversely correlated with prehospital glucose administration per 100 000 population (*r* = −0.331, *p* < 0.05) and per 1000 EMS patients (*r* = −0.425, *p* < 0.05); the inverse correlation per 1000 patients with diabetes was not statistically significant (*r* = −0.274, *p* = 0.062). Although diabetologist density was positively correlated with insulin prescribing proportion (*r* = 0.326, *p* < 0.05), it was inversely correlated with prehospital glucose administration.

**Conclusions:**

Marked geographic disparities in prehospital glucose administration for hypoglycemia were observed in Japan. Higher diabetologist density was associated with lower rates of prehospital glucose administration, despite a higher proportion of insulin prescribing, supporting efforts to improve access to certified diabetologists and strengthen diabetologist–primary care collaboration.

## Graphical abstract

Among older adults in Japan, higher diabetologist density was associated with lower rates of hypoglycemic events recognized by Emergency Medical Services (EMS) requiring prehospital glucose administration, despite higher insulin prescribing. These findings support improved access to certified diabetologists and stronger diabetologist–primary care collaboration.

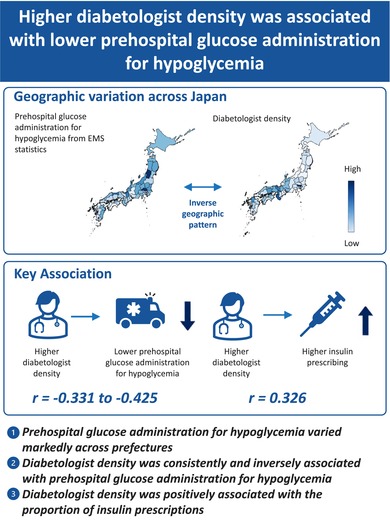

## 1 Introduction

Severe hypoglycemia is a critical adverse event in diabetes treatment and is consistently associated with poor prognosis, including increased risks of cardiovascular disease, cognitive impairment, depressive symptoms, falls, and frailty [1, 2, 3, 4, 5, 6]. Older adults are considered a high‐risk population of severe hypoglycemia, and the Japan Geriatrics Society and the Japan Diabetes Society issued the Elderly Diabetes Clinical Practice Guidelines 2023 to guide individualized treatment in this group [7]. However, our previous research revealed suboptimal adherence to these guidelines in real‐world settings, such as overdosing of sulfonylureas (SUs) in older adults [8], which may increase the risk of hypoglycemia [9].

Regional variation in diabetes‐related mortality and the quality of diabetes care has been reported in Japan, and these differences appear to be associated with area‐level socioeconomic conditions and aspects of healthcare resources and service delivery [10, 11]. However, evidence linking regional differences in diabetes care to regional hypoglycemia incidence remains limited. The availability of diabetologists may be one relevant resource. First, diabetologists may reduce hypoglycemia risk through more appropriate prescribing and treatment individualization [12, 13, 14]. Second, effective collaboration between diabetologists and primary care physicians (PCPs) is important for mitigating this risk and improving the quality of regional diabetes care. A systematic review reported improved glycemic control in integrated care models in which diabetologists actively collaborate with PCPs [15].

However, publicly available data from the Japan Diabetes Society indicate that Japan has only approximately 7000 certified diabetologists [16], which is insufficient to cover all patients with diabetes [14]. In addition, an uneven distribution of diabetologists across prefectures is expected, consistent with reports that specialists in various medical fields tend to be concentrated in urban areas [17, 18, 19]. Accordingly, many patients with diabetes may be managed by PCPs, and this tendency may be stronger in regions with limited access to diabetologists. Consequently, the geographic maldistribution of diabetologists may contribute to regional differences in the incidence of severe hypoglycemia via differences in diabetes management and diabetologist–PCP collaboration.

Therefore, this study aimed to describe geographic disparities in prehospital glucose administration for hypoglycemia recorded in Emergency Medical Service (EMS) statistics across Japan, as an EMS‐recognized indicator related to severe hypoglycemia, and to explore its association with the distribution of diabetologists. We also assessed regional prescribing patterns by diabetologist density to explore potential mediating factors.

## 2 Methods

### 2.1 Study Design and Data Source

This ecological cross‐sectional study was conducted across all 47 prefectures in Japan to evaluate the associations among diabetologist density, prehospital glucose administration for hypoglycemia, and regional prescribing patterns. We used three main data sources: EMS statistics from the Fire and Disaster Management Agency (FDMA), the publicly available diabetologist list of the Japan Diabetes Society, and medication history data from the Musubi electronic medication history database used in community pharmacies across Japan.

### 2.2 Prehospital Glucose Administration by Paramedics

This study used prehospital glucose administration by paramedics as an indicator of EMS‐recognized hypoglycemic events, rather than as a measure of the overall incidence of hypoglycemia. EMS data were obtained from national statistics, which are collected annually by the FDMA via a standardized electronic reporting system across all Japanese prefectures [20]. These statistics include information such as age, gender, severity, reason for emergency call, and paramedic interventions, including the administration of glucose solution.

The Japanese EMS system is operated by local fire and rescue agencies, ensuring nationwide coverage with paramedics dispatched in response to a 119 call [21]. In April 2020, the scope of paramedic interventions was expanded through regulatory revisions that permitted the administration of glucose solution during hypoglycemic attacks following blood glucose measurement [22]. We used EMS statistics for the year 2022, which reflect this expanded scope of intervention. The analysis was restricted to individuals aged 65 years and over.

To account for variations in population size and underlying disease prevalence, we calculated prefecture‐level rates of prehospital glucose administration using three distinct standardization methods: (1) the number of glucose administrations per 100 000 population in each prefecture [23], (2) the number of glucose administrations per 1000 diabetes patients in each prefecture [24], and (3) the number of glucose administrations per 1000 EMS patients recorded in the EMS statistics.

We additionally obtained prefecture‐level numbers of active paramedics and paramedics certified for glucose administration from the 2024 FDMA report [25] and expressed them per 100 000 population for supplementary analyses.

### 2.3 Diabetologist Density

Diabetologist density was defined as the number of certified diabetologists per 100 000 population in each prefecture. Information on certified diabetologists was obtained from the publicly available specialist list on the Japan Diabetes Society website as of July 2025 [16]. Population denominators were derived from official prefectural population statistics [23]. Diabetologist density was calculated separately for each prefecture and used for mapping and correlation analyses.

### 2.4 Prescribing Patterns (Musubi Dataset)

In this study, we analyzed prescribing patterns using Musubi, a cloud‐based electronic medication history system used by more than 15% of community pharmacies in Japan. Musubi stores detailed prescription information, including age, gender, medication name, prescription date, duration, and dosage. A previous report suggested that Musubi data have a reasonable degree of national representativeness with respect to patient age and sex distribution [8].

For the present analyses, we extracted records for patients aged 65 years or older who were prescribed at least one glucose‐lowering medication, including SUs, dipeptidyl peptidase‐4 (DPP‐4) inhibitors, biguanides (e.g., metformin), sodium–glucose cotransporter 2 (SGLT2) inhibitors, glucagon‐like peptide‐1 (GLP‐1) receptor agonists, glinides, thiazolidinediones, and insulin, from community pharmacies that had implemented Musubi and agreed to provide data for this study, between January 2019 and June 2025. This extended observation window was used to improve the stability of prefecture‐level estimates of prescribing patterns. At the prefectural level, we calculated (1) the proportion of each medication class and the proportion of concomitant glucose‐lowering medications, and (2) the proportion of insulin prescriptions among all glucose‐lowering prescriptions. Among these indicators, only the prefecture‐level proportion of insulin prescriptions was subsequently mapped.

### 2.5 Statistical Analysis

Descriptive statistics (mean ± standard deviation) were calculated for continuous variables, and frequencies (percentages) were calculated for categorical variables. The Musubi dataset was first stratified into the top five and bottom five prefectures according to diabetologist density. This stratification was used for exploratory comparison of patient characteristics between regions with relatively high and low diabetologist density. Comparisons between these two groups were performed using Student's *t*‐tests for continuous variables and chi‐square tests for categorical variables.

To assess prefecture‐level associations between diabetologist density and key outcomes, we calculated Pearson correlation coefficients (*r*) at the prefectural level. The correlation analysis included the three standardized rates of prehospital glucose administration for hypoglycemia and supplementary EMS personnel indicators. As an exploratory analysis, we examined the association between diabetologist density and the prefecture‐level proportion of insulin prescriptions derived from the Musubi database. We did not conduct mediation analysis due to the cross‐sectional design and the limited number of observational units (prefectures).

All statistical analyses were performed using R version 4.3.2 (R Foundation for Statistical Computing, Vienna, Austria). Two‐sided *p* values < 0.05 were considered statistically significant.

## 3 Results

### 3.1 Regional Prehospital Glucose Administration for Hypoglycemia

**FIGURE 1:**
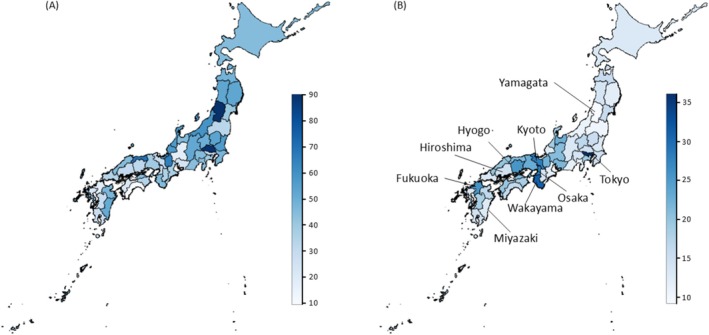
Prefecture‐level prehospital glucose administration for hypoglycemia and diabetologist density in Japan. (A) Number of glucose administrations per 100 000 population in 2022, based on national Emergency Medical Service statistics. (B) Number of certified diabetologists per 100 000 population in each prefecture as of July 2025, based on the specialist list of the Japan Diabetes Society.

**FIGURE 2:**
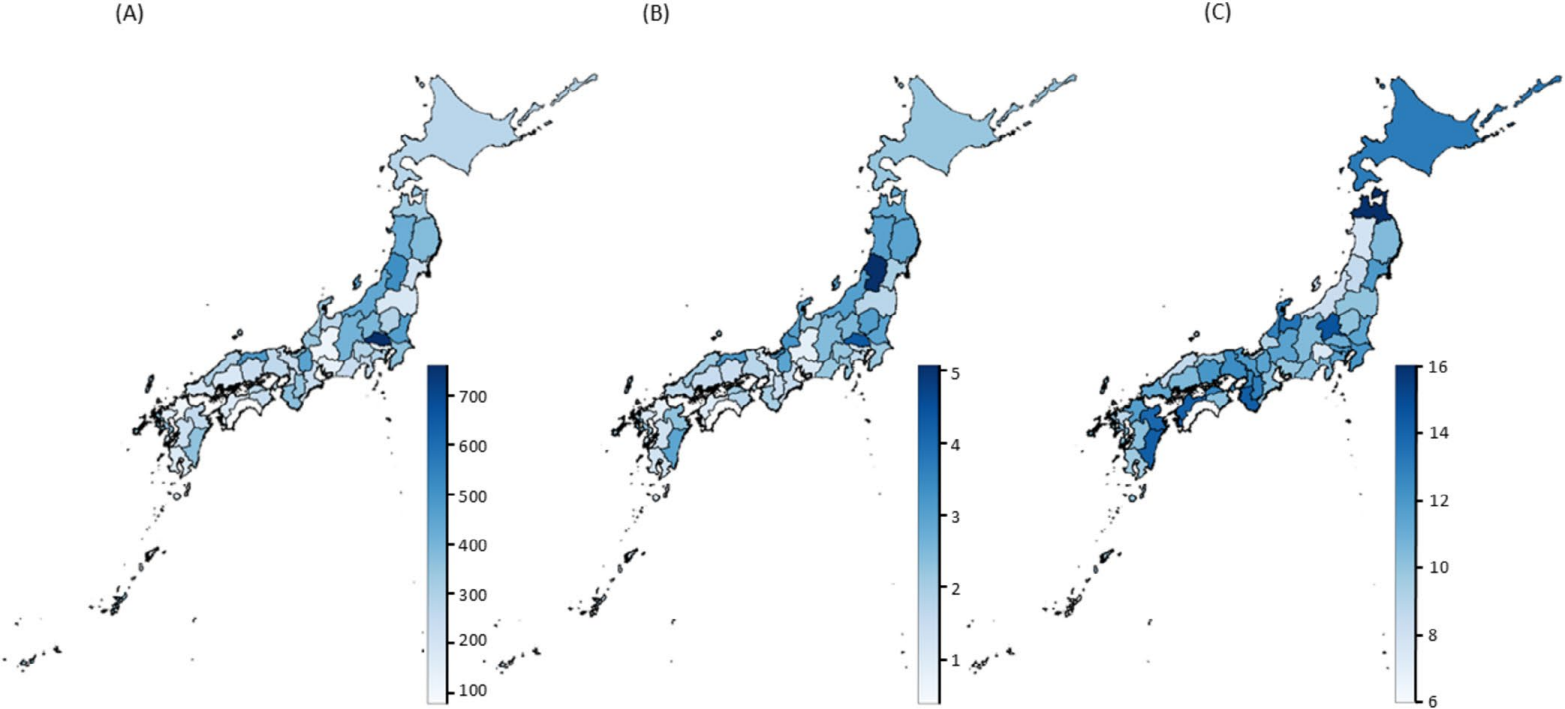
Prefecture‐level standardized rates of prehospital glucose administration for hypoglycemia and insulin prescribing in Japan. (A) Number of glucose administrations per 1000 diabetes patients in 2022. (B) Number of glucose administrations per 1000 Emergency Medical Service (EMS) reports in 2022. (C) Prefecture‐level proportion of insulin prescriptions among all glucose‐lowering prescriptions, based on Musubi data from January 2019 to June 2025. (A and B) include all 47 prefectures; (C) includes 46 prefectures because insulin prescribing data were not available for Okinawa.

In 2022, national EMS statistics recorded 9816 instances of prehospital glucose administration, of which 7196 occurred in adults aged 65 years or older. Across prefectures, all three standardized rates demonstrated marked geographic variation in prehospital glucose administration for hypoglycemia (Table 1, Figures 1A and 2A,B).

**TABLE 1:** Prefecture‐level rates of prehospital glucose administration for hypoglycemia, insulin prescribing, and diabetologist density in Japan, 2022.

Prefecture	EMS statistics			Musubi database	Certified diabetologist list
	Number of prehospital glucose administrations per 100 000 population	Number of prehospital glucose administrations per 1000 EMS patients	Number of prehospital glucose administrations per 1000 diabetes patients	Proportion of insulin prescriptions, %	Diabetologist density (per 100 000 population)
Hokkaido	44.5	2.2	273.0	12.9	13.0
Aomori	48.0	2.8	322.6	17.4	15.6
Iwate	52.8	2.9	377.2	10.3	12.0
Miyagi	40.5	2.0	216.1	12.1	15.6
Akita	52.7	3.0	417.8	7.5	13.7
Yamagata	90.0	5.1	507.8	8.8	9.1
Fukushima	32.8	1.8	179.4	10.2	10.4
Ibaraki	56.7	3.0	466.7	11.6	12.8
Tochigi	53.4	3.0	288.7	10.1	15.4
Gunma	49.2	2.5	386.7	14.8	16.6
Saitama	89.6	4.3	760.8	11.0	14.3
Chiba	46.9	2.2	357.8	12.2	14.6
Tokyo	30.2	1.2	166.0	12.1	36.1
Kanagawa	39.0	1.6	287.4	11.4	19.2
Niigata	58.9	3.0	437.1	8.5	10.7
Toyama	45.7	2.3	271.4	13.6	22.5
Ishikawa	56.8	3.0	320.0	13.2	18.6
Fukui	57.0	3.2	343.6	9.6	12.3
Yamanashi	44.7	2.2	282.5	7.2	13.8
Nagano	51.8	2.4	403.6	10.4	13.1
Gifu	17.6	0.8	107.1	11.5	20.7
Shizuoka	44.0	2.2	240.8	10.4	11.5
Aichi	23.8	1.0	135.9	10.1	21.5
Mie	36.3	1.5	237.0	10.3	11.7
Shiga	65.5	2.9	452.7	11.4	22.1
Kyoto	27.6	1.2	236.4	12.0	28.0
Osaka	29.3	1.1	210.1	12.7	27.7
Hyogo	39.3	1.8	259.0	12.5	22.8
Nara	35.2	1.5	346.5	13.1	13.7
Wakayama	45.3	2.0	363.2	14.0	31.2
Tottori	69.3	3.2	476.9	9.8	21.2
Shimane	44.5	2.1	288.6	8.9	19.8
Okayama	31.9	1.5	237.7	12.0	25.5
Hiroshima	30.7	1.5	176.9	10.6	14.4
Yamaguchi	34.2	1.7	191.5	11.3	13.1
Tokushima	34.6	1.8	242.9	10.1	18.3
Kagawa	43.2	2.1	270.8	12.8	20.9
Ehime	21.8	1.0	171.4	14.1	19.7
Kochi	9.5	0.4	76.7	6.3	16.1
Fukuoka	17.2	0.8	104.2	11.8	25.7
Saga	22.2	1.1	147.4	8.7	13.1
Nagasaki	44.6	2.1	346.4	9.8	14.5
Kumamoto	28.6	1.3	222.5	10.2	18.3
Oita	46.7	2.3	253.6	14.0	16.5
Miyazaki	50.7	2.9	356.0	14.4	12.5
Kagoshima	28.2	1.3	170.1	9.6	14.3
Okinawa	46.9	1.8	356.5	—	18.0

*Note:* Prehospital glucose administration rates are based on EMS statistics for 2022; insulin prescribing is based on Musubi prescription data from January 2019 to June 2025; diabetologist density is based on the certified diabetologist list as of July 2025.

Abbreviation: EMS, Emergency Medical Services.

When expressed as glucose administrations per 100 000 population, the highest rates were observed in Yamagata (90.0 per 100 000), Saitama (89.6), Tottori (69.3), Shiga (65.5), and Niigata (58.9). In contrast, the lowest rates were observed in Kochi (9.5 per 100 000), Fukuoka (17.2), Gifu (17.6), Ehime (21.8), and Saga (22.2). Thus, the rate in Yamagata, the prefecture with the highest rates, was approximately 10‐fold greater than that in Kochi, the prefecture with the lowest rate, underscoring the pronounced prefecture‐level heterogeneity in prehospital glucose administration for hypoglycemia (Figure S1A–C).

### 3.2 Regional Diabetologist Density

As of July 2025, the Japan Diabetes Society had certified 7106 diabetologists nationwide. When expressed as the number of certified diabetologists per 100 000 population, diabetologist density showed marked geographic variation across prefectures (Table 1 and Figure 1B). The five prefectures with the highest densities were Tokyo (36.1 per 100 000 population), Wakayama (31.2), Kyoto (28.0), Osaka (27.7) and Fukuoka (25.7). In contrast, the five prefectures with the lowest densities were Yamagata (9.1 per 100 000 population), Fukushima (10.4), Niigata (10.7), Shizuoka (11.5), and Mie (11.7), indicating more than a four‐fold difference between the highest and lowest prefectures (Figure S1D).

### 3.3 Regional Prescribing Patterns

In the Musubi database, over 30 million prescription records were available during the study period. Of these, 678 501 patients aged 65 years or older who were prescribed at least one glucose‐lowering medication were included in the analysis.

Patient characteristics and overall prescribing patterns are presented in Table 2. The table also shows patient characteristics stratified by diabetologist density, comparing patients residing in the top five prefectures with the highest diabetologist density (Tokyo, Wakayama, Kyoto, Osaka, and Fukuoka) and the bottom five prefectures (Yamagata, Fukushima, Niigata, Shizuoka, and Mie).

**TABLE 2:** Characteristics and glucose‐lowering medication use among patients aged ≥ 65 years, by diabetologist density.

	All (*N* = 678 501)^a^	Top 5 prefectures^b^ (*N* = 160 989)	Bottom 5 prefectures^c^ (*N* = 67 547)	*p* ^d^
Age (years), mean ± SD	76.9 ± 7.5	77.0 ± 7.5	76.5 ± 7.6	< 0.001
Age ≥ 65 years, *n* (%)	678 501 (100.0)	160 989 (100.0)	67 547 (100.0)	NA
Age ≥ 75 years, *n* (%)	390 259 (57.5)	93 318 (58.0)	36 645 (54.3)	< 0.001
Female, *n* (%)	300 337 (44.3)	71 458 (44.4)	29 734 (44.0)	0.108
Glucose‐lowering medications, *n* (%)				
DPP‐4i	464 568 (68.5)	109 100 (67.8)	47 779 (70.7)	< 0.001
SU	109 120 (16.1)	27 184 (16.9)	10 712 (15.9)	< 0.001
Glimepiride	88 144 (13.0)	20 955 (13.0)	9212 (13.6)	< 0.001
Gliclazide	20 367 (3.0)	6228 (3.9)	1257 (1.9)	< 0.001
Glibenclamide	4127 (0.6)	938 (0.6)	443 (0.7)	0.042
Biguanide	257 689 (38.0)	60 823 (37.8)	25 866 (38.3)	0.021
SGLT2i	315 652 (46.5)	73 345 (45.6)	30 292 (44.8)	0.002
α‐Gi	74 627 (11.0)	15 247 (9.5)	7864 (11.6)	< 0.001
GLP‐1RAs	54 926 (8.1)	14 627 (9.1)	4303 (6.4)	< 0.001
GLP‐1/GIP RA	7920 (1.2)	2093 (1.3)	523 (0.8)	< 0.001
Thiazolidione	11 892 (1.8)	2513 (1.6)	1341 (2.0)	< 0.001
Imeglimin	17 962 (2.6)	4311 (2.7)	1664 (2.5)	0.004
Glinide	57 622 (8.5)	12 934 (8.0)	5111 (7.6)	< 0.001
Insulin	78 814 (11.6)	19 777 (12.3)	6807 (10.1)	< 0.001
With SU	7371 (1.1)	1859 (1.2)	711 (1.1)	0.037
With DPP‐4i	35 255 (5.2)	8508 (5.3)	3464 (5.1)	0.128
With GLP‐1RA	13 682 (2.0)	3751 (2.3)	860 (1.3)	< 0.001
With GLP‐1/GIP RA	2485 (0.4)	635 (0.4)	148 (0.2)	< 0.001
Number of concomitant glucose‐lowering medications, mean ± SD	1.90 ± 1.03	1.90 ± 1.04	1.82 ± 0.99	0.012

*Note:* NA, not applicable (age ≥ 65 years was an inclusion criterion).

Abbreviations: α‐GI, α‐glucosidase inhibitor; DPP‐4i, dipeptidyl peptidase‐4 inhibitor; GLP‐1 RA, glucagon‐like peptide‐1 receptor agonist; GLP‐1/GIP RA, GLP‐1/glucose‐dependent insulinotropic polypeptide receptor agonist; SGLT2i, sodium–glucose cotransporter 2 inhibitor; SU, sulfonylurea.

^a^ Values are presented as mean ± standard deviation or *n* (%), as appropriate.

^b^ Top five prefectures: Tokyo, Wakayama, Kyoto, Osaka, and Fukuoka (highest diabetologist density).

^c^ Bottom five prefectures: Yamagata, Fukushima, Niigata, Shizuoka, and Mie (lowest diabetologist density).

^d^ *p* values compare the top five and bottom five prefectures (Student's *t*‐test for continuous variables and *χ* ^2^ test for categorical variables).

### 3.4 Regional Comparison of Diabetologist Density and Prehospital Glucose Administration

Across prefectures, the three standardized rates of prehospital glucose administration for hypoglycemia, mapped in Figures 1A and 2A,B, showed broadly similar geographic patterns. An inverse geographic pattern was apparent when these incidence maps were compared with the diabetologist density map (Figure 1B). Prefectures with higher diabetologist density, such as Tokyo, Kyoto, Fukuoka, and Hyogo (darker shading in Figure 1B), generally exhibited lower rates of prehospital glucose administration (lighter shading in Figures 1A and 2A,B), whereas prefectures with lower diabetologist density tended to have higher rates.

**FIGURE 3:**
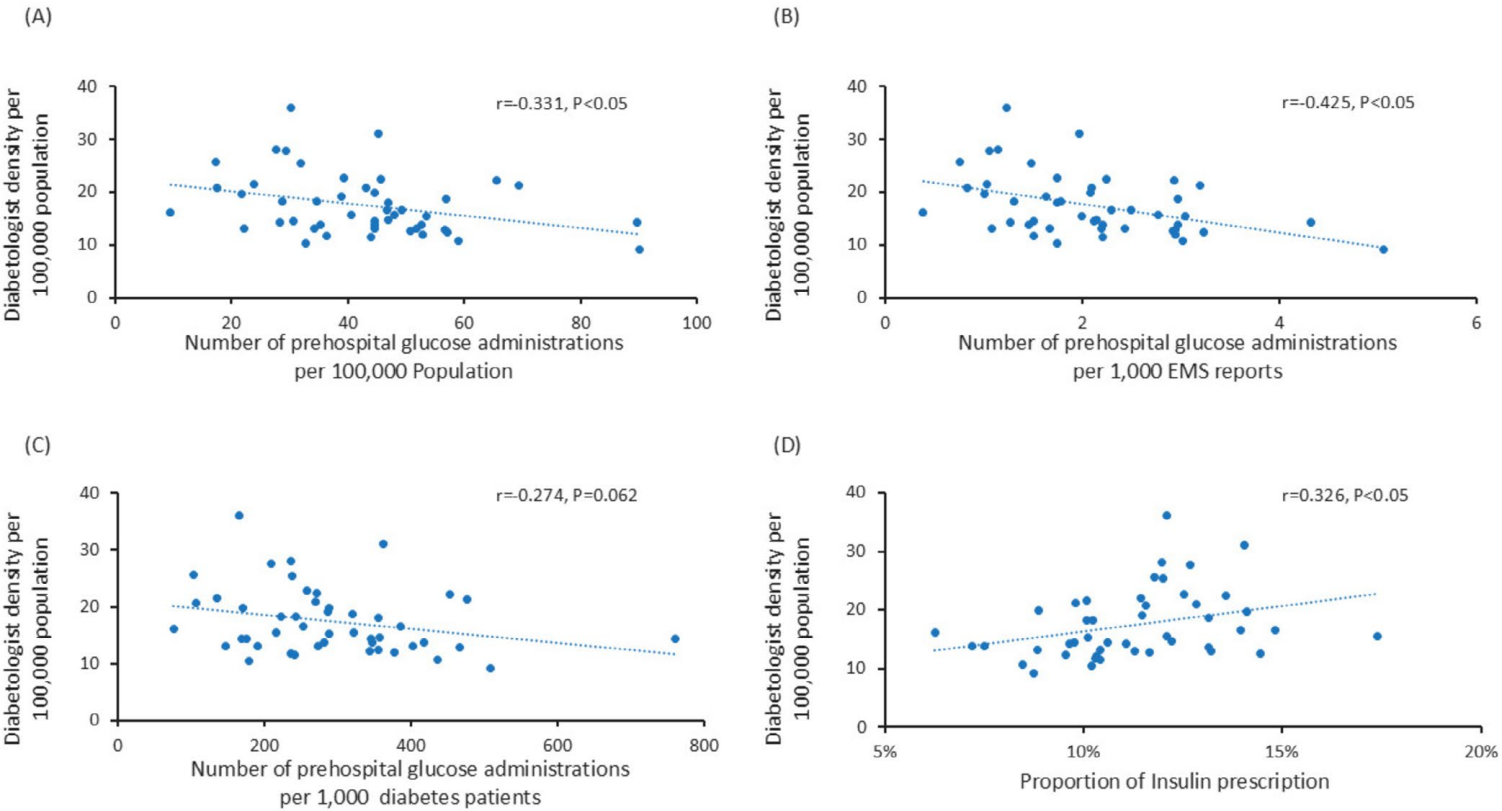
Correlations between diabetologist density, standardized rates of prehospital glucose administration for hypoglycemia, and insulin prescribing at the prefectural level. (A) Diabetologist density versus the number of prehospital glucose administrations per 100 000 population in 2022. (B) Diabetologist density versus the number of prehospital glucose administrations per 1000 Emergency Medical Service (EMS) reports in 2022. (C) Diabetologist density versus the number of prehospital glucose administrations per 1000 diabetes patients in 2022. (D) Diabetologist density versus the prefecture‐level proportion of insulin prescriptions among all glucose‐lowering prescriptions, based on Musubi data from January 2019 to June 2025. Pearson correlation coefficients (*r*) and *p*‐values are shown in each panel.

This inverse pattern was confirmed in Pearson correlation analyses at the prefectural level (Figure 3). Diabetologist density was significantly and inversely correlated with the rate of prehospital glucose administration per 100 000 population (*r* = −0.331, *p* < 0.05; Figure 3A) and per 1000 EMS patients (*r* = −0.425, *p* < 0.05; Figure 3B). The correlation with the rate per 1000 diabetes patients was also negative but did not reach statistical significance (*r* = −0.274, *p* = 0.062; Figure 3C). In contrast, diabetologist density was positively and significantly correlated with the prefecture‐level proportion of insulin prescriptions (*r* = 0.326, *p* < 0.05; Figure 3D). Supplementary analyses showed no significant correlations between EMS personnel indicators and the three standardized rates of prehospital glucose administration for hypoglycemia (Tables S1 and S2).

## 4 Discussion

This nationwide ecological study integrated EMS statistics on prehospital glucose administration, diabetologist density, and real‐world prescribing data to quantitatively examine how regional differences in diabetologist density relate to prehospital glucose administration for hypoglycemia among older adults in Japan. We identified three main findings: (1) prehospital glucose administration for hypoglycemia varied markedly across prefectures; (2) diabetologist density was consistently and inversely associated with standardized rates of prehospital glucose administration for hypoglycemia; and (3) diabetologist density was positively associated with the proportion of insulin prescriptions. Taken together, these findings suggest that geographic variation in the availability of certified diabetologists may be associated with EMS‐recognized hypoglycemic events and the safety of diabetes care at the population level.

We observed substantial regional disparities in prehospital glucose administration for hypoglycemia, with approximately tenfold differences between the highest (e.g., Yamagata) and lowest (e.g., Kochi) prefectures. This indicates that EMS‐recognized hypoglycemic events requiring prehospital glucose administration vary substantially across Japan. The marked geographic variation observed in the present study differs from findings from the J‐DOME Registry [26], which did not observe significant geographic differences in diabetes quality‐of‐care indicators—such as glycated hemoglobin levels, regular ophthalmic examinations, and urinary albumin measurements—based on data from 2938 patients across 116 medical facilities. However, the J‐DOME Registry used seven broad regional blocks, whereas the present study examined all 47 prefectures. Urbanized and sparsely populated rural areas often coexist [27]. Such broad aggregation may mask local variation in healthcare processes and outcomes, consistent with reports of marked heterogeneity in healthy life expectancy and healthcare resources across secondary medical areas in Japan [28]. Nevertheless, because prehospital glucose administration captures only a subset of hypoglycemic events and may be influenced by bystander intervention, EMS activation, transport time, and local protocols, these findings do not establish regional differences in the overall incidence of severe hypoglycemia.

Maldistribution of diabetologists may be one of the factors explaining disparities in prehospital glucose administration for hypoglycemia. This is consistent with evidence that diabetologist involvement is associated with more guideline‐concordant and individualized care than primary care alone [12, 13, 14]. In a nationwide survey, most patients with type 2 diabetes were managed by general practitioners, whereas only about one‐fifth were under the care of diabetologists [14]. Therefore, the observed geographic variation in prehospital glucose administration may reflect not only differences in direct access to certified diabetologists but also indirect, system‐level influences related to how diabetologists are embedded within regional care networks and support PCPs through guidance, education, referral pathways, and shared treatment protocols.

Diabetologist density may also contribute to regional prescription trends. Although insulin is a major pharmacological risk factor for severe hypoglycemia in older adults, diabetologists may be more likely than general practitioners to initiate and titrate insulin with structured education and close follow‐up, which could mitigate hypoglycemia risk [12, 13]. Accordingly, in regions with higher diabetologist density, greater insulin use may reflect safer implementation of insulin therapy, including patient education, dose adjustment, and monitoring, whereas regions with fewer diabetologists may have less specialist support for safe insulin use or may rely more on agents such as SUs. These interpretations remain speculative given the absence of patient‐level data but are consistent with the observed ecological pattern of higher insulin prescribing and lower rates of prehospital glucose administration for hypoglycemia in higher‐density prefectures.

From a clinical and policy perspective, lower diabetologist density may be associated with a higher burden of hypoglycemic events requiring EMS intervention among older adults with diabetes. Efforts to improve access to certified diabetologists in underserved areas, together with strengthening structured diabetologist–primary care collaboration, may help enhance the safety of glucose‐lowering therapy.

This study has several limitations. First, because the analysis was conducted at the prefectural level, ecological fallacy is possible. Second, causal inference is not possible because of the cross‐sectional design. Third, we did not conduct mediation analysis given the cross‐sectional ecological design and the limited number of analytical units (prefectures). Fourth, prehospital glucose administration captures only a small subset of hypoglycemic events. Kurahashi et al. reported that only 2.0% of ambulance‐transported patients with hypoglycemia received prehospital glucose administration [29]. This indicator may also be affected by bystander response, EMS activation, transport time, and local protocols. Therefore, our findings should not be taken as the overall incidence of hypoglycemia or severe hypoglycemia. Fifth, prefecture‐level analyses may not fully capture within‐prefecture heterogeneity, particularly between urban centers and remote rural areas. Sixth, diabetologist density was derived from certification records and did not account for actual clinical time, practice location within prefectures, or contributions from other diabetes care professionals. Seventh, prescribing patterns were based on the Musubi database, which covers a subset of community pharmacies and may not be fully representative. Selection of participating pharmacies and prefecture‐level differences in Musubi penetration may have influenced regional prescribing estimates; in addition, the prescribing period (2019–2025) did not perfectly align with the EMS data year (2022). Finally, we lacked patient‐level data on key confounders, such as diabetes duration, comorbidities, renal function, and socioeconomic status, which limits the interpretation and underscores the need for future individual‐level studies to confirm and extend these ecological associations.

Despite these limitations, this study has several strengths. We integrated nationwide EMS statistics, prefecture‐level diabetologist density, and real‐world prescribing data to quantify geographic variation in EMS‐recognized hypoglycemic events requiring prehospital glucose administration among older adults. The concordant findings across multiple standardized rates strengthen the robustness of the observed associations.

## 5 Conclusion

In conclusion, we found marked prefecture‐level disparities in EMS‐recognized hypoglycemic events requiring prehospital glucose administration among older adults in Japan. Higher diabetologist density was associated with lower rates of these events and higher insulin prescribing. These findings support improving access to certified diabetologists and strengthening diabetologist–primary care collaboration in underserved areas.

## Author Contributions

**Tomoya Saika:** conceptualization; writing – original draft. **Yuki Kado:** formal analysis. **Michiko Yamazaki:** writing – review and editing. **Tohru Takebe:** writing – review and editing. **Masaya Sakamoto:** writing – review and editing. **Atsushi Nakagomi:** writing – review and editing. All authors read and approved the final manuscript.

## Funding

The authors have nothing to report.

## Ethics Statement

This study was approved by the institutional review board of the International University of Health and Welfare, Japan (approval number: 25‐MT‐017), and was carried out according to the principles of the Declaration of Helsinki (as revised in Fortaleza, Brazil, October 2013).

## Consent

The institutional review board of International University of Health and Welfare determined that the requirement for informed consent could be waived owing to the terms of the contract with pharmacies, which included the use of *Musubi* data for research purposes and the prior anonymization of the data for research purposes.

## Conflicts of Interest

Tomoya Saika, Yuki Kado, Michiko Yamazaki, and Tohru Takebe are employees of KAKEHASHI Inc. Masaya Sakamoto has received lecture fees from Eli Lilly Japan K.K., Novo Nordisk Pharma Ltd., Tanabe Pharma Corporation, Boehringer Ingelheim Seiyaku Co. Ltd., MSD K.K., Daiichi Sankyo Co. Ltd., and Sumitomo Pharma Co. Ltd., and has also received a consulting fee from KAKEHASHI Inc. Atsushi Nakagomi has received research funding from Novo Nordisk Pharma Ltd. and Iwabuchi Pharmaceutical Co. Ltd.

## Supporting information


**Figure S1:** Prefecture‐level rates of prehospital glucose administration for hypoglycemia and diabetologist density in Japan. (A) Number of glucose administrations per 100 000 population. (B) Number of glucose administrations per 1000 diabetes patients in 2022. (C) Number of glucose administrations per 1000 Emergency Medical Service (EMS) patients in 2022. (D) Number of certified diabetologists. Bars are ordered from lowest to highest across the 47 prefectures. The range across prefectures was approximately 10‐fold for (A–C) and approximately fourfold for (D). Dashed lines indicate the mean.
**Table S1:** Prefecture‐level density of active paramedics and paramedics certified for glucose administration in Japan.
**Table S2:** Correlations of paramedic density with prehospital glucose administration rates and diabetologist density.

## Data Availability

The data that support the findings of this study are derived from public domain resources (national EMS statistics published by the Fire and Disaster Management Agency and the publicly available specialist list of the Japan Diabetes Society). The Musubi electronic medication history data are not publicly available due to third‐party restrictions and were accessed under license/contract for the purpose of this study.
