# Supplementary material for: Prefecture‐Level Prehospital Glucose Administration for Hypoglycemia and Diabetologist Density Among Older Adults in Japan: A Nationwide Ecological Study
Source: Geriatr Gerontol Int. 2026 Jul 9;26(7):e70639. doi: 10.1111/ggi.70639 (PMC13348015; doi:10.1111/ggi.70639)
Supplement: Supplementary file 1 — Figure S1: Prefecture‐level rates of prehospital glucose administration for hypoglycemia and diabetologist density in Japan. (A) Number of glucose administrations per 100 000 population. (B) Number of glucose administrations per 1000 diabetes patients in 2022. (C) Number of glucose administrations per 1000 Emergency Medical Service (EMS) patients in 2022. (D) Number of certified diabetologists. Bars are ordered from lowest to highest across the 47 prefectures. The range across prefectures was approximately 10‐fold for (A–C) and approximately fourfold for (D). Dashed lines indicate the mean. Table S1: Prefecture‐level density of active paramedics and paramedics certified for glucose administration in Japan. Table S2: Correlations of paramedic density with prehospital glucose administration rates and diabetologist density. [file GGI-26-0-s001.docx]

**Supplementary material**

**Supplementary Figure S1.** Prefecture-level rates of prehospital glucose administration for hypoglycemia and diabetologist density in Japan. (A) Number of glucose administrations per 100,000 population. (B) Number of glucose administrations per 1,000 diabetes patients in 2022. (C) Number of glucose administrations per 1,000 Emergency Medical Service (EMS) patients in 2022. (D) Number of certified diabetologists. Bars are ordered from lowest to highest across the 47 prefectures. The range across prefectures was approximately tenfold for panels (A)–(C) and approximately fourfold for panel (D). Dashed lines indicate the mean.


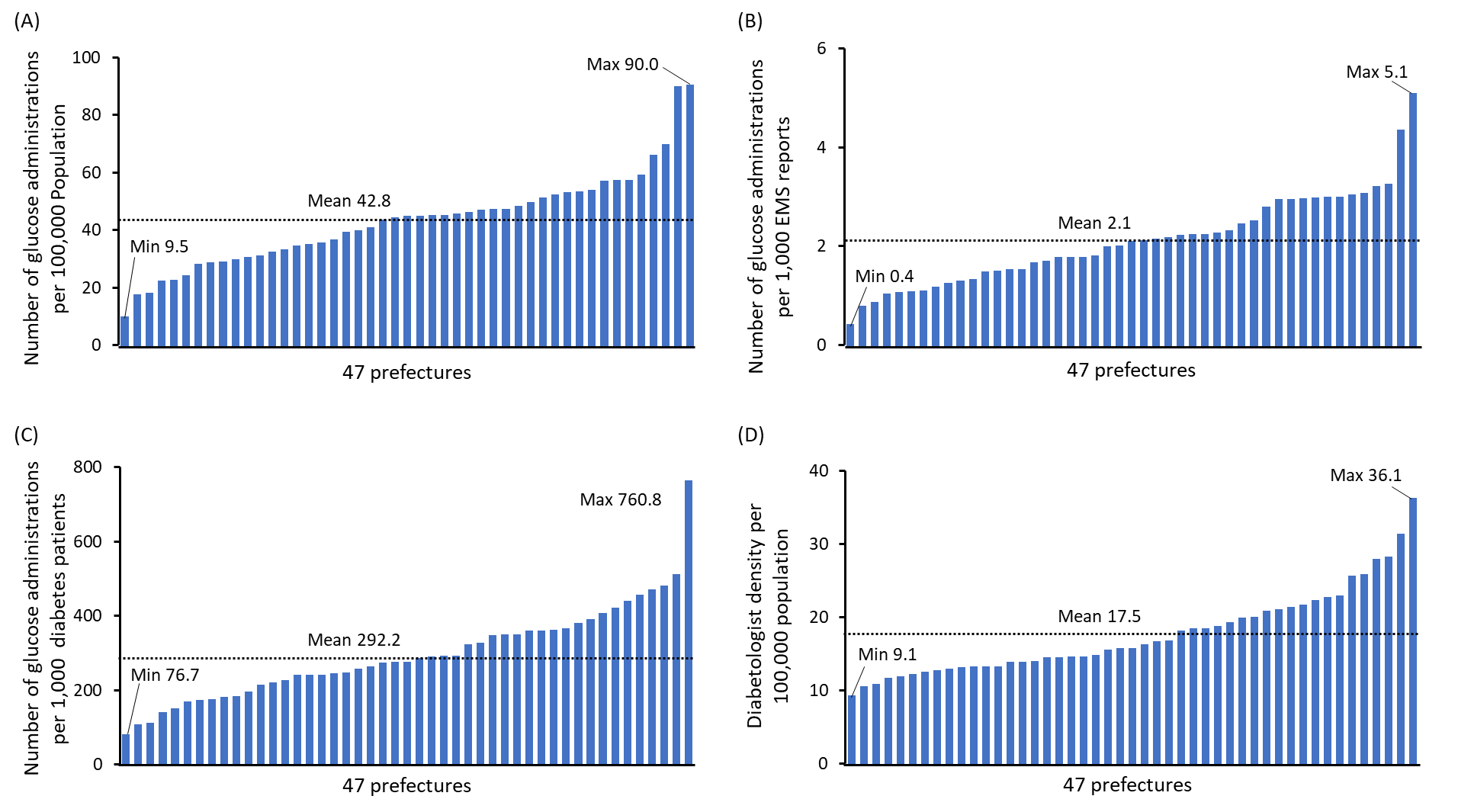


**Supplementary Table S1.** Prefecture-level density of active paramedics and paramedics certified for glucose administration in Japan.

| **Prefecture** | **Active paramedics per 100,000 population** | **Paramedics certified for glucose administration per 100,000 population** |
| --- | --- | --- |
| Hokkaido | 140.7 | 132.4 |
| Aomori | 124.5 | 123.7 |
| Iwate | 112.0 | 112.0 |
| Miyagi | 78.5 | 77.2 |
| Akita | 112.9 | 114.0 |
| Yamagata | 91.1 | 91.1 |
| Fukushima | 97.3 | 97.1 |
| Ibaraki | 111.9 | 117.0 |
| Tochigi | 98.3 | 92.0 |
| Gunma | 87.9 | 74.9 |
| Saitama | 66.2 | 65.8 |
| Chiba | 69.1 | 68.3 |
| Tokyo | 70.1 | 64.2 |
| Kanagawa | 70.3 | 68.2 |
| Niigata | 104.0 | 106.3 |
| Toyama | 81.4 | 73.9 |
| Ishikawa | 99.4 | 82.8 |
| Fukui | 104.3 | 103.8 |
| Yamanashi | 103.6 | 112.3 |
| Nagano | 104.0 | 104.9 |
| Gifu | 105.8 | 85.4 |
| Shizuoka | 70.8 | 71.3 |
| Aichi | 75.9 | 68.4 |
| Mie | 106.0 | 109.6 |
| Shiga | 89.5 | 87.4 |
| Kyoto | 73.0 | 70.9 |
| Osaka | 71.6 | 63.0 |
| Hyogo | 79.4 | 72.7 |
| Nara | 78.3 | 77.5 |
| Wakayama | 103.0 | 90.2 |
| Tottori | 102.8 | 110.1 |
| Shimane | 122.5 | 117.6 |
| Okayama | 88.1 | 84.8 |
| Hiroshima | 83.8 | 82.5 |
| Yamaguchi | 83.2 | 85.8 |
| Tokushima | 96.3 | 96.7 |
| Kagawa | 82.1 | 87.4 |
| Ehime | 83.0 | 84.8 |
| Kochi | 134.7 | 136.8 |
| Fukuoka | 55.8 | 50.7 |
| Saga | 89.7 | 87.7 |
| Nagasaki | 84.1 | 82.1 |
| Kumamoto | 79.0 | 80.1 |
| Oita | 93.1 | 94.7 |
| Miyazaki | 72.1 | 70.4 |
| Kagoshima | 116.4 | 118.1 |
| Okinawa | 152.3 | 148.6 |

Values are expressed as the number of paramedics per 100,000 population in each prefecture.

In these tables, paramedics refers to emergency life-saving technicians in the Japanese EMS system.

**Supplementary Table S2.** Correlations of paramedic density with prehospital glucose administration rates and diabetologist density.

|  | **Active paramedics per 100,000 population** | **Paramedics certified for glucose administration per 100,000 population** |
| --- | --- | --- |
| **Number of prehospital glucose administrations per 100,000 population** | r = 0.08  p=0.597 | r = 0.12  p=0.424 |
| **Number of prehospital glucose administrations per 1,000 EMS reports** | r = 0.10  p=0.513 | r = 0.14  p=0.356 |
| **Number of prehospital glucose administrations per 1,000 diabetes patients** | r = 0.06  p=0.694 | r = 0.11  p=0.479 |
| **Diabetologist density** | r = -0.29  p < 0.05 | r = -0.39  p < 0.05 |

Values are Pearson correlation coefficients (r) and P-values calculated at the prefecture level. EMS, Emergency Medical Services.

In these tables, paramedics refers to emergency life-saving technicians in the Japanese EMS system.
